# ASCP explores the cancer biomarker testing navigator as a novel role to improve laboratory operations and workflows: A special report from the ASCP Biomarker Testing Navigator Project Team

**DOI:** 10.1093/ajcp/aqaf028

**Published:** 2025-05-07

**Authors:** Lynnette Pineault, Karla Valencia, Jennifer Buhay, Alexandra Brown, Suzanne Ziemnik, Melissa Kelly, James Morgante, Ananya Datta Mitra, Lindsay Yanamura, Marie Gilliland, Amy Ferea, Joseph Kim

**Affiliations:** HealthPartners, Bloomington, MN, United States; The University of Texas MD Anderson Cancer Center, Houston, TX, United States; American Society for Clinical Pathology, Chicago, IL, United States; American Society for Clinical Pathology, Chicago, IL, United States; American Society for Clinical Pathology, Chicago, IL, United States; American Society for Clinical Pathology, Chicago, IL, United States; American Society for Clinical Pathology, Chicago, IL, United States; Department of Pathology, University of California Davis Health, Sacramento, CA, United States; Department of Pathology, University of California Davis Health, Sacramento, CA, United States; Spartanburg Regional Healthcare System, Spartanburg, SC, United States; Spartanburg Regional Healthcare System, Spartanburg, SC, United States; Q Synthesis LLC, Newtown, PA, United States

**Keywords:** biomarker testing navigator, cancer biomarker testing, ASCP, quantitative needs assessment, qualitative focus groups, feasibility pilot

## Abstract

**Objective:**

Cancer biomarker testing is a critical element in precision oncology, guiding treatment decisions and improving patient outcomes. However, the complexity and variability of biomarker testing processes present significant challenges for cancer centers, often leading to delays and inefficiencies that can compromise care quality. The American Society for Clinical Pathology explored the concept of a novel laboratory professional role: the cancer biomarker testing navigator (BTN).

**Methods:**

This study explored the feasibility and impact of the BTN role on laboratory operations and workflows through a 3-phase project consisting of a quantitative needs assessment, qualitative focus group discussions, and a short-term feasibility pilot conducted at 2 cancer centers.

**Results:**

The needs assessment revealed that many laboratories lack dedicated staff for coordinating biomarker testing, leading to operational inefficiencies. The roundtable discussions highlighted common challenges in biomarker testing and identified potential benefits of the BTN role, such as improved communication, better tracking of send-out tests, and enhanced task efficiency. The feasibility pilot demonstrated that BTNs could coordinate multigene next-generation sequencing panels and expedite key steps to ensure optimal preanalytical processes, reduce delays in testing, and smooth operations. The BTN role represents a feasible and beneficial addition to pathology laboratories that addresses key operational challenges in cancer biomarker testing and offers a promising solution to streamline laboratory operations, improve multidisciplinary communication, and enhance patient care coordination.

**Conclusions:**

Further exploration is warranted to refine the BTN role and assess its long-term sustainability in and impact on diverse laboratory settings.

**Key Points**

- The cancer BTN is a novel role designed to streamline cancer biomarker testing operations and enhance care quality.
- To explore the feasibility of the BTN role, the ASCP conducted a project that consisted of a quantitative needs assessment, qualitative focus group discussions, and a short-term feasibility pilot.
- The BTN role may be a beneficial addition to pathology laboratories that addresses key operational challenges in cancer biomarker testing.

## INTRODUCTION

Cancer biomarker testing—the analysis of biological markers such as tumor protein expression and somatic genomic alterations—is a key component of precision oncology, guiding treatment decisions, prognosis, and patient stratification. The landscape of prognostic and predictive cancer patient biomarker testing is rapidly expanding. Experts predict a substantial increase in the role of biomarker testing in precision medicine cancer treatment over the next decade.^1-3^ Negative outcomes are currently associated with less-than-optimal biomarker testing rates and efficiencies compared with recommended best practices. For example, studies show that almost half of patients with non–small cell lung cancer do not undergo biomarker testing as recommended by National Comprehensive Cancer Network guidelines. Of the patients with non–small cell lung cancer who do receive biomarker testing, less than one-third receive recommended targeted therapies.^4,5^

Biomarker testing failure rates further exacerbate the issue. Literature reveals that 10% to 20% of biomarker tests are unsuccessful,^4,6,7^ and as many as 90% of these failures may be attributed to preanalytic factors.^4^ Challenges with specimen collection, adequacy, fixation, test ordering, triage, and reporting have all been reported in the literature.^6,8-10^

Inconsistent or incorrect biomarker testing is problematic, and the complexity and variability of biomarker testing processes pose major challenges for pathology laboratory teams and clinicians.^11^ Some of these challenges include the lack of dedicated, skilled professionals and nonstandardized workflows, insufficient communication and coordination, inadequate quality assurance, and limited access to resources and expertise.^12^ These factors can lead to delays, errors, and inefficiencies in biomarker testing that compromise the quality and timeliness of care delivery and may negatively affect patient outcomes.

As the options for cancer biomarkers expand, opportunities exist to improve the operational processes and systems that support biomarker testing in pathology laboratories.^4^ Some laboratory teams have already begun to find ways to address gaps in their own institutions.^13^ This concept of developing specialized roles dedicated to improving patient experience through coordination and expertise is not new. For example, genetic counselors have emerged as valuable contributors on the laboratory team, providing expert consultation, pedigree analysis, and result interpretation while also helping reduce costs through improved test utilization.^14-17^ Although some genetic counselors have roles supporting somatic biomarker testing, most continue to specialize in hereditary/germline testing, leaving an opportunity for a laboratory role specific for cancer biomarker testing.^17-19^

With this in mind, the American Society for Clinical Pathology (ASCP) launched the Biomarker Testing Navigator (BTN) project to explore the following question: What if there were a laboratory professional role serving as a “biomarker testing navigator” to help improve processes, promote best practices, and ensure timely and appropriate cancer biomarker testing? Through this project, the following feasibility study was developed.

## METHODS

The aim of the ASCP BTN feasibility study was to clarify and decipher the applicability of an emerging role for streamlining personalized care for patients with cancer in diverse practice settings by supporting and coordinating comprehensive solutions to address site-specific biomarker testing challenges.

A BTN Project Advisory Committee comprising pathology laboratory leaders guided the ASCP BTN project team. The advisory committee outlined potential key functions of a BTN for exploration. Recognizing that the roles and responsibilities of a BTN may vary across institutions and practice settings, the ASCP BTN project team identified key areas where a BTN may reduce errors, streamline processes, and make major contributions to improve cancer biomarker testing. The advisory committee and project team aimed to answer the following questions:

- Are laboratory team members currently coordinating complex tasks involved in cancer biomarker testing triage? If so, what are their current roles?
- How may cancer biomarker testing operations in the laboratory workflow would be affected if a role dedicated to these tasks were to exist?
- Would it be feasible to create and implement a more formalized BTN role in pathology laboratories?

The study was conducted in 3 phases: a quantitative needs assessment, a series of qualitative roundtable discussions, and a short-term feasibility pilot with 2 cancer centers.

### Quantitative needs assessment survey

A needs assessment survey was designed to collect information about how cancer biomarker testing and triage are performed in various settings and to explore what laboratory staff currently coordinate and manage for cancer biomarker testing processes. Using email and social media, the ASCP invited recipients to participate in this online survey if they were involved in cancer biomarker testing. The survey was open from April 2023 through May 2023 and administered by email to a national sample drawn from ASCP membership. Survey questions are provided in Supplementary Appendix A.

### Qualitative focus groups

Based on initial interest expressed through the BTN needs assessment survey, the ASCP invited a subset of respondents to participate in 1 of 3 virtual focus groups held in June 2023. These focus groups explored how the role of a BTN could be incorporated into the laboratory to facilitate cancer biomarker testing processes. Participants included pathologists and laboratory professionals from diverse practice settings, including academic medical centers; community hospitals; and private practices from rural, suburban, and urban locations. Each 60-minute focus group explored the expectations and preferences of the participants regarding the BTN role, including potential tasks and functions, required qualifications and skills, and implementation and evaluation strategies. Sessions were recorded, and qualitative thematic analysis was performed on the transcriptions. Focus group questions are listed in Supplementary Appendix B.

### Short-term feasibility pilot

Two health care organizations were invited to participate in a 6-month pilot program that ran from May 2023 through Oct 2023. The pilot was designed to explore the feasibility and acceptability of the BTN role and measure its impact on biomarker testing operations. Study sites were chosen based on interest in the pilot, resources available to devote to participation, diversity of practice setting, and biomarker testing requests received. Spartanburg Regional Healthcare System (SRHS), an integrated health care system in South Carolina, studied a BTN supporting somatic solid tumor biomarker testing workflows. University of California (UC) Davis Health, an academic medical center in California, piloted a BTN focused on facilitating hematopoietic cancer biomarker testing requests.

Pilot sites were provided a job description for the BTN developed by the ASCP project team, with an overview of the study protocols and data recording requirements. Each site identified a laboratory professional who had prior knowledge of biomarker testing workflows, including ordering protocols, triage, and other laboratory tasks related to biomarker testing. Laboratory professionals assigned the BTN role recorded biomarker testing volumes, types of tests ordered, time spent on predefined tasks, turnaround time for results, and defects identified throughout the testing workflow. In addition, pilot sites qualitatively evaluated the impact of the BTN on overall workplace and staff efficiency and effectiveness.

## RESULTS

### Quantitative needs assessment survey

The BTN needs assessment survey to ASCP members generated 47 responses. TABLE 1 provides a breakdown of respondents. A Fisher exact test was conducted to examine the association between having dedicated laboratory staff to coordinate and manage biomarker testing and responses to the biomarker testing processes and workflow statements. The Fisher exact test was used due to the limited number of respondents and the likelihood of small, expected frequencies in the contingency tables between items. All analyses were conducted using R, version 4.3.1, statistical software (R Foundation for Statistical Computing). Scale items were aggregated into 2 categories—agree and disagree—by collapsing agreement and disagreement.

When asked whether their laboratory had dedicated staff responsible for coordinating and managing cancer biomarker tests, 30 (65%) reported they did not. In 16 cases, 14 respondents with dedicated staff provided the position title for the staff member, which are provided in Table 2. Of those 16 cases, 12 respondents also described what they perceived as the greatest benefit of having dedicated staff in this role. Processing and tracking responsibilities emerged thematically as being particularly beneficial for having dedicated staff; these benefits are presented in Table 3 with representative quotes.

**Table 2:** Common Roles for Cancer Biomarker Testing Staff

Job title/role	
Pathologist secretary Secretary Histotechnician/histotechnologist Immunohistochemistry technician or scientist Laboratory assistant Pathologists’ assistant Medical laboratory technician	Medical laboratory scientist Molecular technologist or scientist Send-out/reference laboratory technician Senior send-out staff Biomarker scientist Laboratory director Molecular genetics pathologist

**Table 3:** Perceived Benefits for Having Dedicated Biomarker Testing Staff

Theme	Representative quotes
Processing	“Faster turnaround time.”
	“Guides molecular testing as well as interpretation needed for pathologists [‘] diagnosis and oncologists in treatment choices.”
	“Organization of the process.”
	“Standard processing and better understanding of tests ordered.”
	“There are three or four trained secretaries that ensure all testing is sent in a timely manner, which is usually the same day that it is ordered.“
	“This person prepares the samples for send out, verifies appropriate paperwork is present. They keep track of who has the tissue blocks and when or if they are returned. They keep track of the reports as well.”
Tracking	“It creates a core group of people (admin + 1 pathologist) to handle any issues coming from oncologists and/or from the external vendors.”
	“Keeping track of what is sent out to which lab.”
	“Sample monitoring and coordination with pathologist.”
	“Technologist[s] are not responsible – frees up their time.”
	“They are resourceful and have time designated to making sure that tracking is done appropriately and thoroughly.”
	“They handle paperwork.”

**Figure 1:**
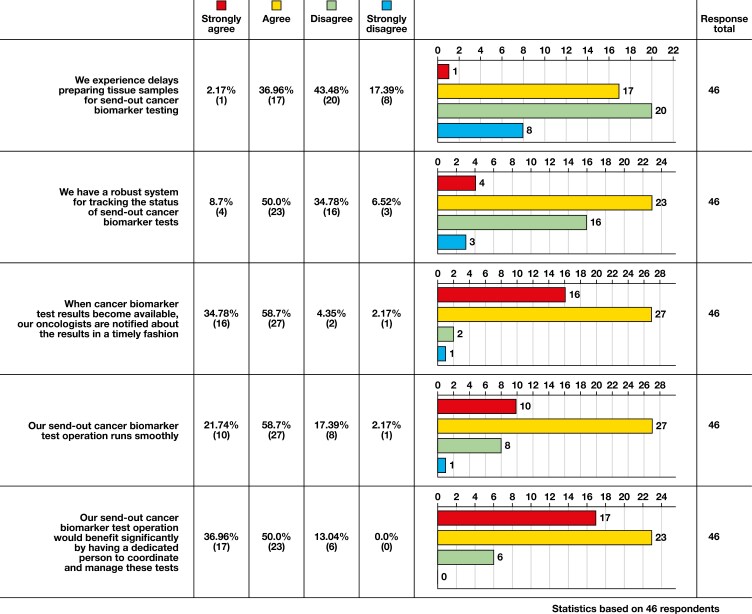
Sample survey responses to the question, “Please rate your agreement with each of the following statements regarding biomarker testing processes/workflow at your institution.”

The benefits of having dedicated staff were further explored through responses to statements regarding biomarker testing processes and workflows. Respondents were asked to rate their agreement with tracking (“We have a robust system for tracking the status of send-out cancer biomarker tests”) and operation (“Our send-out cancer biomarker test operation runs smoothly”) statements using a 4-point Likert-type response scale (ie, strongly disagree, disagree, agree, strongly agree). These responses are illustrated in Figure 1.

Having a dedicated staff member was strongly associated with having a robust system for tracking the status of send-out cancer biomarker tests (*P* = .031) and a send-out cancer biomarker test operation that runs smoothly (*P* = .018). Although further research is needed to provide more insight into these associations, the initial results from the needs assessment suggest that dedicated staff may play a crucial role in certain aspects of the biomarker testing workflow, particularly in tracking and operational efficiency.

### Qualitative focus groups

The ASCP invited 9 pathologists and 7 laboratory professionals to participate in 1 of 3 virtual focus group discussions. An average of 5 people participated in each meeting. A qualitative thematic analysis of virtual focus group discussion transcripts revealed common themes with the challenges and opportunities related to biomarker testing in different practice settings. The participants discussed various aspects of biomarker testing, such as test ordering, specimen handling, test performance, result reporting, and communication. Some of the common issues they faced were delays due to insurance authorization, insufficient tissue samples, lack of standardization and integration of systems, and variability in oncologist preferences in test ordering and reference laboratory selection.

The focus group discussions also explored the feasibility of a BTN role responsible for the oversight and coordination of key biomarker testing processes, from test ordering to result reporting. Some respondents reported that their laboratories already had dedicated staff to coordinate biomarker test orders. These roles emerged because of the complexity and time required to manage a growing number of orders that need to be sent to outside reference laboratories for testing. Participants envisioned that the BTN could have a similar degree of oversight. Participants indicated that the BTN would need strong administrative and logistical skills, some scientific knowledge pertaining to biomarker testing, and communication skills to collaborate with pathologists, oncologists, and other stakeholders. Benefits and barriers to the BTN role were also discussed, as were metrics that could be used to demonstrate the effectiveness of such a role. Focus group discussion topics and discussion summary are provided in Table 4.

**Table 4:** Focus Group Topics and Discussion Summary

Topic	Discussion summary
Operational challenges	Insufficient tissue samples Incomplete/incorrect order information Lack of standardization and integration of systems Variability in oncologist preferences and protocols Delays due to insurance authorization Difficulty tracking the status of send-out tests Interfaces with different information technology systems Long turnaround times for multigene next-generation sequencing panels Manual result processing steps for noninterfaced laboratory tests
Optimal processes for timely and comprehensive testing	Multidisciplinary oversight for testing policies and processes Test orders aligned with guideline recommendations Reflex orders when appropriate Optimized turnaround times Perform more testing in-house whenever possible Digital pathology
Potential skills of a BTN	Working knowledge of cancer biomarker testing Operational, administrative and logistical skills Strong communication and collaboration skills Responsible for oversight and coordination of the biomarker testing process, from test ordering to result reporting
BTN feasibility considerations	Metrics to support the role include reduction in delays in test ordering, improving turnaround time for results, and decreasing incorrect test orders (wait and cycle times) The feasibility and cost justification for a BTN may vary depending on the organization and its specific needs A dedicated role may not be realistic if testing volumes are low Would private practice laboratories be able to provide financial justification for this role? Consider combining the responsibilities of this role with other positions, such as a send-out test coordinator or a prior authorization specialist Role may be justified by nonfinancial metrics, such as patient and clinician satisfaction

Abbreviation: BTN, biomarker testing navigator.

### Short-term feasibility pilots

Spartanburg Regional Healthcare System and UC Davis Health participated in the short-term pilots exploring how a BTN could streamline cancer biomarker testing workflows and facilitate key steps in the testing process. They also subjectively assessed the impact of the BTN on overall workplace and staff efficiency and effectiveness.

#### Spartanburg Regional Healthcare System

Spartanburg Regional Healthcare System is an integrated health care system in the northern region of South Carolina that includes Gibbs Cancer Center & Research Institute, a comprehensive cancer center that provides medical oncology, radiation oncology, and surgical oncology services. The Spartanburg Medical Center laboratory precision medicine team, which facilitates biomarker test requests for Gibbs and other locations within the system, participated in the pilot and assigned a staff member to be the designated BTN for 6 months.

At SRHS, most biomarker testing orders are for next-generation sequencing, and specimens are sent to external reference laboratories for testing. The Spartanburg precision medicine team built on their prior work of streamlining workflows for send-out testing and explored the potential for a BTN to ensure that the correct tests are ordered, confirm optimal tissue fixation, and monitor result turnaround times. The biggest benefit of the BTN demonstrated during this pilot was the centralization of biomarker testing information and communication in the laboratory. The BTN received test orders, reviewed pathology reports, accessed patient records and insurance details, requested specimens, and prepared them for testing. Incorporating the BTN role reduced the need for histotechnologists, who would typically perform these tasks, to coordinate and manage send-out testing requests.

The pilot BTN tracked 233 send-out cancer biomarker tests ordered from June 2023 to September 2023, recording defects in 5.6% of the orders, correcting them before sending samples to the appropriate reference laboratory for testing. The pilot BTN also documented common causes for send-out biomarker test delays. Tables 5 and 6 list the most common errors and delays.

**Table 5:** Spartanburg Regional Healthcare System Biomarker Order Defects Reported

Order defects
Incorrect test type ordered Incorrect specimen type requested Incorrect specimen date provided Incorrect insurance information provided Incorrect order used Reflex testing not performed Incorrect tumor type reported

**Table 6:** Biomarker Testing Delays

Order testing delays
Inpatient hold (waiting 14 d after inpatient discharge) Waiting for tissue to be received from outside storage location/archived storage Awaiting insurance prior authorization Waiting for appropriate diagnosis coding (payor requirements) Reference laboratory receiving limitations (eg, Friday send-out restrictions)

Some current Spartanburg workflows for biomarker testing were refined during the pilot. A mechanism to provide real-time testing status updates to oncologists was developed, and physician notification was added through the electronic health record (EHR) system when results are finalized, when possible. Following the pilot, Spartanburg expanded the pathology team to include 2 BTNs and a supervisor, further reducing the biomarker coordination burden of histology staff and increasing support for precision pathology workflows for SRHS.

#### UC Davis Health

The UC Davis Comprehensive Cancer Center is a National Cancer Institute–Designated Comprehensive Cancer Center serving Central Valley and inland Northern California. The hematopathology team at UC Davis piloted the BTN role by focusing on bone marrow tests performed on patients with various hematologic malignancies. Under the leadership of an academic hematopathologist, a bone marrow technician was assigned to the pilot BTN role and worked with the hematopathologists and hemato-oncologists to streamline the biomarker testing process for bone marrow samples. In this pilot, the pilot BTN supported the workflow from sample collection through the receipt of final biomarker test results. The pilot BTN worked with attending physicians and fellows to develop standard testing protocols for specific types of hematologic malignancies, and orders were reviewed, triaged, and placed at the time of biopsy.

Sample adequacy and biomarker test ordering was tracked for 218 bone marrow biopsies scheduled from July 2023 to October 2023. Biomarker testing was performed on 208 of these patients, resulting in 844 biomarker tests/panels ordered. The remaining 12 biopsies were either cancelled or samples were determined to be insufficient for testing.

**Figure 2:**
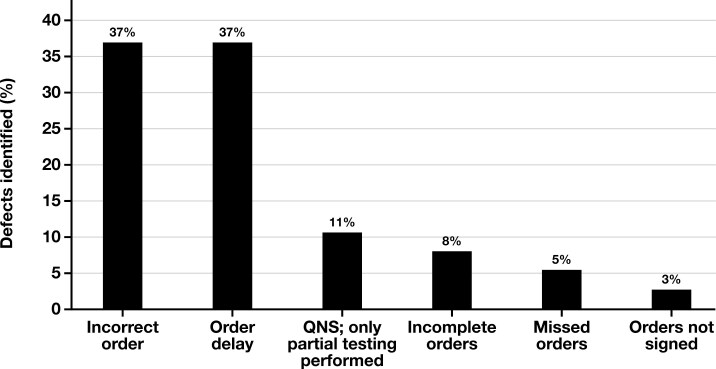
Defects identified. QNS indicates quantity not sufficient.

Of the 208 patients receiving biomarker testing, 30 (14%) had defects that delayed testing, with 4 (2%) of these having multiple defects. The most common defects the pilot BTN identified were delays in receiving orders once the sample had been collected and incorrect orders, either because the wrong order was placed in the EHR or a laboratory was requested that did not perform the testing desired. When biopsy samples had low yield or were insufficient, the pilot BTN coordinated with ordering clinicians and pathologists to prioritize or cancel tests. The pilot BTN also identified instances when requested testing was incomplete and results pending and situations when reports were uploaded incorrectly in the EHR or not uploaded at all. The most common defects are shown in Figure 2.

During the pilot, standardized bone marrow order sets were created for acute lymphoblastic leukemia, acute myeloid leukemia, chronic lymphocytic leukemia, chronic myeloid leukemia, myelodysplastic syndrome, multiple myeloma, and a few specific types of lymphoma. The order sets indicate which tests are performed in house and which require send-out to an external reference laboratory. This approach simplified the test ordering process and helped manage expectations for complex send-out tests that have longer turnaround times. The team at UC Davis used this pilot to assess send-out testing volume to justify the expansion of in-house test offerings, resulting in plans to incorporate several new in-house molecular tests. The team also explored the role of integrations between electronic health records, laboratory information systems, and reference laboratories to facilitate test ordering and resulting.

## DISCUSSION

This feasibility study illustrates the complexity of cancer biomarker testing and the many touchpoints where defects and delays can occur and negatively affect result turnaround times and patient treatment. Although other mitigation strategies, such as tumor boards, clinical pathways, standardized order sets, and clinical decision support, exist, numerous operational challenges persist.

More than one-third (37%) of needs assessment participants experienced delays triaging tissue samples for cancer biomarker orders, revealing unnecessary time spent coordinating and facilitating biomarker testing, especially when multiple reference laboratories are required. More than half of needs assessment participants (65%) do not have dedicated staff to oversee, coordinate, or manage cancer biomarker testing, and the majority (87%) agree that they would benefit considerably from such a role. Other studies have shown how laboratories experience delays triaging tissue samples for biomarker cancer testing,^20^ and responses provided during the focus group discussions for this feasibility study further substantiate these findings.

These defects can be mitigated through enhanced collaboration between pathology and clinical care teams to drive practice and performance improvement initiatives supported by a BTN role. This mitigation was demonstrated during the health system pilots, where participating laboratory teams found that they could catch and correct defects sooner with the use of a BTN role, allowing for processes to be refined and improved as insights were gained into the causes of the defects. Supporting this concept, a 2021 article published in this journal suggests the “creation of a laboratory navigator system” to assist pathologists as their roles in biomarker testing increase.^21^ As mentioned previously, some institutions have already created dedicated roles (eg, “precision medicine stewards”) who manage test orders, streamline prior authorizations and other precatalytic requirements, and monitor the completion of cancer biomarker testing results.^22^

The diverse staffing models observed in the pilot programs and some surveyed groups underscore the critical need for specialized expertise to support biomarker testing pathways and a clear delineation of roles and responsibilities. This feasibility study suggests that a BTN would be most impactful in an oversight role, using advanced knowledge to provide guidance and oversight for test selection and protocol development while also keeping pace with new and evolving biomarker tests and their corresponding treatment implications. Tasks that are more clerical or administrative in nature can be managed by a dedicated biomarker testing navigation team or existing laboratory support/processing staff.

Cancer biomarker testing is an important step in identifying patients who may benefit from targeted therapies or immunotherapy across multiple types of cancers.^23^ As ongoing research advances new cancer treatment options, successful translation of this evidence into clinical practice relies on effective operational workflows that ensure that patients receive recommended testing and treatment. Patient-derived pathology samples are precious resources, and the handling and triage of tissue, cells, and other samples is best done by pathologists and trained laboratory professionals who can ensure that the right sample is submitted for the right test at the right time, decreasing defects and delays and bridging the quality chasms that exist in cancer biomarker testing to deliver high-quality cancer care.^24^

### Limitations

Although the number of needs assessment survey respondents was limited, the participants represented diverse practice areas and population settings, and the results aligned with the findings from the focus groups and pilot studies.

Focus group participants acknowledged the value a BTN could bring to cancer biomarker testing workflows and also highlighted several challenges that could hinder successful implementation of the role. Although metrics such as delays and defect reduction and an increase in patient and clinician satisfaction could help justify a BTN, many felt that feasibility and cost justification for a BTN may vary depending on the organization and its specific needs. It would be beneficial to explore the financial impact of the BTN role on cost avoidance from the reduction of unnecessary testing and the potential for downstream revenue as a result of increased productivity for oncologists and pathologists, drawing parallels to the influence that roles such as pathologists’ assistants and cytologists have had on pathology and cytology workflows.^25-28^

The BTN pilot studies were conducted at 2 health systems that were evaluating different biomarker specimen pathways for solid tumor and hematopoietic samples. Although both organizations have programs to support underserved populations, findings may not be representative of smaller practices or encompass all marginalized communities. Other laboratories may need to consider how the BTN role could be tailored to their needs, based on factors such as volume, test order mix, and how frequently external reference laboratories are used.

Given the short-term scope of the project, the ASCP did not capture other metrics to quantify the extent to which the BTN may overlap other roles or departments (internal or external to the laboratory) or if additional responsibilities exist that could be assumed by the BTN to further optimize workflows. In addition, this study did not encompass other credentialed individuals, such as laboratory professionals and genetic counselors, who may possess the necessary skills and expertise to help orchestrate and coordinate of these functions.

### Future directions

Beyond the aforementioned advantages, a BTN may provide an unexpected solution to workforce staffing challenges and shortages. In 2021, the ASCP and the University of Washington Center for Health Workforce Studies collaborated on a publication outlining the needs of the clinical laboratory workforce. This study indicated that although the demand for laboratory testing is increasing, the number of people qualified to perform laboratory testing is decreasing.^29^ In addition, participant feedback collected from the ASCP continuing education programs indicates many laboratory roles are not optimally aligned with current responsibilities. At the same time, the United States is facing a shortage of physicians, with some suggesting that new methods of health care delivery using a team-based approach as a viable solution.^30-32^ Developing roles such as the BTN that can coordinate and facilitate care, streamlining workflows, and reducing redundancies and delays may prove essential in these new care delivery models.

Based on the findings from this project and the evaluation of current literature supporting the need for a more formalized BTN role, the ASCP is developing (at the time of manuscript submission) an online certificate program to educate and train laboratory staff who may desire work in roles that support biomarker navigation. The certificate program will be developed to encapsulate real-world experiences and provide meaningful education and other supporting resources to current and future laboratory professionals working as cancer biomarker testing navigators. The ASCP also intends to develop resources, such as job descriptions and work aids, to provide guidance for organizations that may want to integrate a BTN into their laboratories, providing an additional level of position control into their staffing models for biomarker testing and precision pathology. More information about this certificate program is expected to be available in early 2025.

The landscape of cancer biomarker testing is rapidly evolving and expanding as new targeted therapies and immunotherapies emerge as standards of care. The BTN is a novel role in the pathology laboratory that can oversee, coordinate, and streamline a range of biomarker testing operations. The ASCP BTN project suggests that the BTN role is a beneficial and feasible solution to improve laboratory workflows and has a positive impact on multidisciplinary team communication, patient care coordination, quality, and efficiency and can be justified through evaluation of the following variables:

- **Reduction of test order defects.** Multiple steps ranging in complexity must be coordinated when send-out biomarker tests are ordered and resulted. Each testing laboratory may have its own unique ordering requirements and pathways. Having a role that can proficiently oversee the navigation of these steps will decrease delays and defects.
- **Task efficiency and staff alignment.** Current team members may be pulled away from the core functions of their roles to support biomarker testing. A BTN can streamline work for clinicians, pathologists, and other laboratory professionals, enabling them to work at the top of their professional scope, while the BTN becomes the subject matter expert for biomarker navigation.
- **Streamlined communication between clinicians.** A BTN can facilitate communication among laboratory staff, pathologists, oncologists, and other members of the health care team to help streamline the coordination of cancer biomarker testing.
- **Improved timeliness of results.** By supporting workflows that intercept defects in the biomarker testing workflow, the BTN contributes to a reduction in testing delays, leading to more timely treatment for patients with cancer.
- **Transforming care from volume to value.** As health care organizations shift care delivery models from volume to value, a BTN enhances high-quality care delivery through the facilitation of biomarker appropriate pathology specimen management and utilization.

The data and insights this feasibility study provides will inform the refinement of the BTN role while aligning professional responsibilities contributory to the laboratory-based precision medicine workforce. Further exploration is warranted to assess the level of knowledge and skills a BTN should possess and whether people in this emerging role could become mainstream members of the pathology team.

## Supplementary material

Supplementary material is available at *American Journal of Clinical Pathology* online.

**Table 1:** Breakdown of Survey Respondents

Survey question	Overall, No. (%) (N = 47)
**What is your primary role in the laboratory?**	
Medical laboratory director	3 (6.4)
Administrator (eg, operations director, manager, supervisor)	5 (10.6)
Pathologist	19 (40.4)
Technologist/scientist (eg, BB, C, CG, CT, H, HTL, M, MB, MLS, MT certifications)	8 (17.0)
Technician (eg, DPT, HT, MLA, MLT, PBT certifications)	8 (17.0)
Other laboratory professional	4 (8.5)
**How would you describe your laboratory practice?**	
University/academic teaching hospital	12 (25.5)
Community hospital-based laboratory	22 (46.8)
Clinical outpatient laboratory	2 (4.3)
Independent private laboratory	4 (8.5)
Reference laboratory	1 (2.1)
Military facility, Veterans Health Administration	2 (4.3)
Other^a^	4 (8.5)
**In what type of area is your laboratory located?**	
Rural	9 (19.1)
Urban	19 (40.4)
Suburban	17 (36.2)
Not sure	2 (4.3)

^a^“Other” includes locums tenens, numerous laboratory environments, physician-owned laboratory, and other laboratory.

aqaf028_suppl_Supplementary_File
