# Supplementary material for: ASCP explores the cancer biomarker testing navigator as a novel role to improve laboratory operations and workflows: A special report from the ASCP Biomarker Testing Navigator Project Team
Source: Am J Clin Pathol. 2025 May 7;163(6):926–35. doi: 10.1093/ajcp/aqaf028 (PMC12159526; doi:10.1093/ajcp/aqaf028)
Supplement: aqaf028_suppl_Supplementary_File [file aqaf028_suppl_supplementary_file.docx]

# Appendix A

**Needs Assessment Survey**

**Q1. How does your laboratory perform most tissue-based cancer biomarker testing for solid tumors? (select one)**

- Performs in-house
- Sends out to a reference lab
- A mix of in-house and send-out testing
- Other (please specify)

**Q2. What type(s) of tissue-based cancer biomarker testing does your laboratory perform in-house for solid tumors? (select all that apply)**

- Immunohistochemistry (IHC)
- In situ hybridization (ISH)
- Polymerase chain reaction (PCR)
- Next-generation sequencing (NGS)
- [Exclusive] None of the above
- Other (please specify)

**Q3. How often does your laboratory send tissue samples (solid tumors) out to reference laboratories for biomarker testing? (select one)**

- Never
- Rarely
- Sometimes
- Frequently

**Q4. At your institution, orders for biomarker tests are usually placed by: (select one)**

- A single group of medical oncologists
- Two groups of medical oncologists
- Three or more groups of medical oncologists

**Q5. How often do oncologists enter a repeat order for biomarker testing because they do not realize that a previous order has already been placed? (select one)**

- Never
- Rarely
- Sometimes
- Frequently

**Q6. How has the volume of NGS test orders (for large multigene panels) changed over the past 12 months at your institution? (select one)**

- Significant increase in NGS test orders
- Moderate increase in NGS test orders
- No changes in NGS test orders
- Moderate decrease in NGS test orders
- Significant decrease in NGS test orders

**Q7. On average, how many different reference labs do you utilize for send-out biomarker testing? Enter number: ____**

**Q8. If you track the turnaround time (TAT) for send-out NGS testing (large multigene panel), what has been the average TAT (from the time the sample is sent to the time the results are obtained)?**

Enter number of days: ___

Skip this question if you do not track TAT for send-out biomarker tests

**Q9. Do you have a person in the laboratory who is responsible for coordinating and managing send-out biomarker tests? (e.g., a send-out test coordinator or a precision medicine test coordinator)? (select one)**

- Yes
- No

If “yes,” then what is the job title for this person? ___

If “yes,” what has been the biggest benefit of having this person on the team? ___

If “no,” then:

**Q10. Has your laboratory explored the possibility of having someone coordinate and manage send-out biomarker tests? (select one)**

- Yes
- No

**Q11. Rate your level of agreement for each of the following statements: (Strongly Agree -> Strongly Disagree; 5 point-scale)**

- **We experience delays preparing tissue samples for send-out biomarker testing**
- **We have a robust system for tracking the status of send-out biomarker tests**
- **When biomarker test results become available, our oncologists are notified about the results in a timely fashion**
- **Our send-out biomarker test operation runs smoothy**
- **Our send-out biomarker test operation would benefit significantly by having a dedicated person coordinate and manage these tests**

***Demographics***

**Q12. How would you describe your laboratory practice? (select one)**

- University/academic teaching hospital
- Community hospital-based laboratory
- Clinical outpatient laboratory
- Independent private laboratory
- Reference laboratory
- Military facility, Veterans Health Administration (VHA)
- Industry/equipment/pharma
- Other (please specify)

**Q13. In what type of area is your laboratory located? (select one)**

- Rural
- Urban
- Suburban
- Not sure

**Q14. What state is your laboratory located? (drop-down list of states)**

**Q15. What is your primary role in the laboratory? (select one)**

- Pathologist
- Administration/management
- Technologist/Scientist (e.g., MLS)
- Technician (e.g., HT, MLT)
- Coordinator
- Other (please specify)

# Appendix B

**Focus Group Questions**

- Tell us about your laboratory and your position/role
- How many different medical oncology groups treat patients diagnosed with cancer?
- When new patients are diagnosed with advanced cancer (solid tumors) and require biomarker testing (e.g., multi-gene panels), what are some of the biggest operational challenges when managing in-house tests vs send-out tests?
- How should biomarker tests be ordered?
- What are the steps for an "optimal" test ordering process?
- Where do things break down when tests are ordered?
- What is the role for “reflex” biomarker testing protocols? What should be included?
- When tissue appears to be very limited for testing, what should be done?
- How should labs track the status of send-out tests?
- What are ways to avoid receiving duplicate test orders?
- What about situations when patients are diagnosed elsewhere (i.e., samples are outside your lab)?
- What are some factors that may cause delays in testing?
- How should results route to the treating oncologist? To the lab? To the patient?
- Imagine having a cancer “biomarker testing navigator” in the lab based on the following description:
  - Oversees day-to-day activities of send-out test operations
  - Coordinates with ordering providers and clinical laboratories to ensure appropriate and timely test ordering and resulting
  - Monitors the quality of testing data by tracking turn-around-times, QNS (quantity not sufficient), and incomplete results
  - Receives and reviews specific testing information to assist clinical staff with appropriate specimen choice for testing
  - Coordinates alternative testing strategies such as liquid biopsies for cases with insufficient tissue quantity
- How do you see this person working in the lab?
- What are the main benefits you may expect to observe?
- What barriers currently exist to make this role work in the lab?
- What types of metrics would you track to assess value and productivity?
- How feasible would it be to have someone in this role?
